# Evaluating peritumoral and intratumoral radiomics signatures for predicting lymph node metastasis in surgically resectable non-small cell lung cancer

**DOI:** 10.3389/fonc.2024.1427743

**Published:** 2024-10-11

**Authors:** Ran Xu, Kaiyu Wang, Bo Peng, Xiang Zhou, Chenghao Wang, Tong Lu, Jiaxin Shi, Jiaying Zhao, Linyou Zhang

**Affiliations:** ^1^ Department of Thoracic Surgery, The Second Affiliated Hospital of Harbin Medical University, Harbin, China; ^2^ The Second Clinical Medical College, Harbin Medical University, Harbin, China; ^3^ Department of Thoracic Surgery, Ruijin Hospital, Shanghai Jiao Tong University School of Medicine, Shanghai, China

**Keywords:** peritumoral, intratumoral, radiomics, lymph node metastasis, non-small cell lung cancer

## Abstract

**Background:**

Whether lymph node metastasis in non-small cell lung cancer is critical to clinical decision-making. This study was to develop a non-invasive predictive model for preoperative assessing lymph node metastasis in patients with non-small cell lung cancer (NSCLC) using radiomic features from chest CT images.

**Materials & methods:**

In this retrospective study, 247 patients with resectable non-small cell lung cancer (NSCLC) were enrolled. These individuals underwent preoperative chest CT scans that identified lung nodules, followed by lobectomies and either lymph node sampling or dissection. We extracted both intratumoral and peritumoral radiomic features from the CT images, which were used as covariates to predict the lymph node metastasis status. By using ROC curves, Delong tests, Calibration curve, and DCA curves, intra-tumoral-peri-tumoral model performance were compared with models using only intratumoral features or clinical information. Finally, we constructed a model that combined clinical information and radiomic features to increase clinical applicability.

**Results:**

This study enrolled 247 patients (117 male and 130 females). In terms of predicting lymph node metastasis, the intra-tumoral-peri-tumoral model (0.953, 95%CI 0.9272-0.9792) has a higher AUC compared to the intratumoral radiomics model (0.898, 95%CI 0.8553-0.9402) and the clinical model (0.818, 95%CI 0.7653-0.8709). The DeLong test shows that the performance of the Intratumoral and Peritumoral radiomics models is superior to that of the Intratumoral or clinical feature model (p <0.001). In addition, to increase the clinical applicability of the model, we combined the intratumoral-peritumoral model and clinical information to construct a nomogram. Nomograms still have good predictive performance.

**Conclusion:**

The radiomics-based model incorporating both peritumoral and intratumoral features from CT images can more accurately predict lymph node metastasis in NSCLC than traditional methods.

## Graphical Abstract

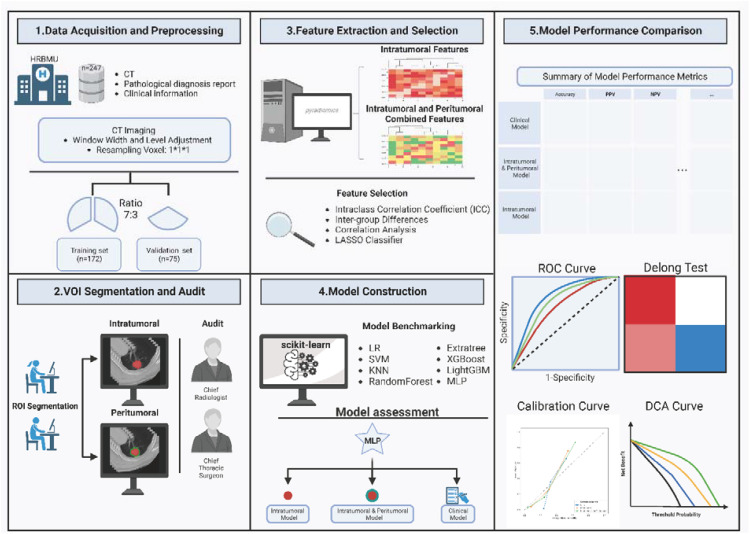

## Introduction

Lung cancer is a prominent cause of cancer-related deaths, with non-small cell lung cancer (NSCLC) accounting for a significant proportion (1). For stages IA to IIIA localized lymph node metastasis, surgery is the primary treatment approach (2); however, for advanced stage IIIB-IV NSCLC that is not resectable through surgery, chemoradiotherapy, immune therapy, or targeted therapy are necessary. Hence, accurate staging is critical for effective NSCLC treatment. Clinical staging typically begins with chest CT scans, which may suggest an indication for lymph node metastasis. Recently, mediastinoscopic lymph node biopsy or endobronchial ultrasound-guided fine needle aspiration have been proposed as essential methods for definitive staging (3). However, these methods are associated with high costs and potential complications. Therefore, there is an urgent need to develop non-invasive methods for preoperative staging of surgically resectable NSCLC and guide the treatment of NSCLC patients. In addition, PET/CT is also considered an important basis for determining staging, but CT is currently the preferred imaging examination for patients with newly treated pulmonary nodules (4, 5), so the model developed using CT images is more universal.

Radiomics is a powerful method that can transform CT images into high-throughput quantifiable data (6). The combination of these radiomic features with machine learning algorithms to construct clinical prediction models is a promising research direction (7). Prior studies have suggested that radiomic features are useful in predicting patient overall survival, pathological response to adjuvant therapy, or lymph node metastasis status in various cancers (8–12), including NSCLC. However, most studies focused only on intratumoral radiomic features and neglected peri-tumoral areas despite their importance in encompassing information related to tumor progression and evolution influenced by interactions between intra-tumoral cells and elements in the peritumoral region, such as lymphatic or vascular invasion and angiogenesis (10, 13–15). Previous radiomics models of lymph node metastasis in non-small cell lung cancer usually only focused on the characteristics of the tumor itself without introducing the characteristics of the surrounding tissue, which may lead to limitations in model performance (16–20). The metastatic status of lymph nodes is not only affected by the characteristics of the primary tumor, such as tumor size, shape, density, etc., but is also related to changes in the tumor microenvironment, including surrounding tissues. Surrounding tissue characteristics can provide important information about the interaction of the tumor with its microenvironment. For example, changes in texture of surrounding tissue, abnormalities in local blood vessel density, irregularities in tumor boundaries, and the degree of tumor infiltration may indicate tumor invasiveness and metastatic potential. Therefore, a comprehensive consideration of radiomic features of surrounding tumor tissues may better characterize tumor invasiveness and metastatic potential. Constructing a comprehensive, non-invasive preoperative radiomic prediction model for lymph node metastasis status in surgically resectable NSCLC is essential.

In this study, we collected CT images and clinical data from 247 patients with surgically resectable NSCLC. We established three preoperative lymph node metastasis prediction models using intratumoral radiomic features, a combination of peritumoral and intratumoral radiomic features, and clinical characteristics. We compared the predictive performance of these models, highlighting the importance of peritumoral radiomic features in predicting lymph node metastasis status. Finally, we constructed a nomogram with wider applicability by fusing the best-performing intratumoral-peritumoral radiomics model and clinical indicators. Our results may provide a new perspective for non-invasive preoperative lymph node diagnosis.

## Materials and methods

### Data acquisitions

The chest CT images, and clinical information of patients used in this study were obtained from our center, and the study was approved by the Ethics Committee (Approval No.: KY2022-144). The acquisition of written informed consent from patients was waived because of the retrospective design. All data usage was performed in accordance with the supervision of the Ethics Committee. The cases included in the study were diagnosed as surgically resectable NSCLC at our hospital between 2019 and 2020. Lymph node metastasis status and pathological diagnosis were determined through postoperative pathological examination. Lymph node dissection strategies for all patients were consistent with current recommendations from The American Association for Thoracic Surgery (21). The exact pathological classification was determined by multiple pathological experts in our center after surgery and a formal medical diagnosis was issued. The detailed inclusion criteria were as follows (1): Patients with pulmonary nodules diagnosed by chest CT; (2) Patients with chest CT images available in Digital Imaging and Communications in Medicine (DICOM) format; (3) Patients who underwent pulmonary lobectomy and systematic lymph node sampling or dissection; (4) Patients with confirmed postoperative histopathology of primary NSCLC with clear lymph node metastasis status for each station; (5) Patients who did not receive neoadjuvant treatment or chemoradiotherapy in the past; (6) Patients who did not have previous thoracic surgery. All CT images were acquired using a 64-channel CT scanner (Discovery 750, GE Healthcare, Milwaukee, USA) with the following scanning parameters: tube voltage of 120kV, tube current of 100-250mAs, layer thickness of 0.625-5 mm, field of view (FOV) of 350-400 mm, 512 x 512 matrix, and reconstructed layer thickness of 0.625-5 mm. The CT images were reconstructed using filtered back-projection (FBP) and adaptive statistical iterative reconstruction (ASIR) at a level of 40% ASIR. The standard kernel was used for the reconstruction. In total, 247 cases were included in the study, with 87 positive and 160 negatives for lymph node metastasis. In addition, we extracted the CT imaging data of lung adenocarcinoma patients (n=219) from two datasets in The Cancer Imaging Archive (TCIA) database for external validation of the radiomics model (https://www.cancerimagingarchive.net/browse-collections/,Collection label: NSCLC-Radiomics-Genomics and NSCLC Radiogenomics).

### CT image data preprocessing and ROI segmentation

To eliminate batch differences between CT image data, all CT data were first adjusted to a window width of -150 and a window level of 1700. The voxel spacing was then adjusted to 1 × 1 × 1 using the nearest interpolation algorithm to account for different scanning parameters and image resolutions (22). Grayscale discretization was performed using a fixed bin width of 25 Hounsfield Units (HU All chest CTs were loaded into ITK-SNAP software (23) (version: 3.8.0) for tumor region segmentation by two thoracic surgeons with over 5 years of clinical experience. The segmented tumor area was examined layer by layer and revised by a chief thoracic surgeon and a radiologist.

### Extraction of radiomic features from VOI

The volume of interest (VOI) comprised intratumoral and extratumoral regions. The intratumoral region was manually segmented layer by layer, while the extratumoral region was obtained by extending 3 voxels around the intratumoral VOI. Radiomic features were extracted using the ‘pyradiomics’ package (24) in Python. To capture a comprehensive set of features, we extracted features under multiple filters, including Original, Wavelet, Square, SquareRoot, Logarithm, Gradient, Exponential, LBP3D, and Laplacian of Gaussian filter (LoG). For the LoG filter, we used sigma values of 1.0, 2.0, and 3.0 to enhance texture recognition for fine and rough textures. Under these filters, we extracted radiomic features from the intratumoral and peritumoral regions, including First Order Features, Shape Features, Gray Level Co-occurrence Matrix (GLCM) Features, Gray Level Zone Size Matrix (GLSZM) Features, Gray Level Run Matrix (GLRLM) Features, Neighbungay Gray Tone Difference Matrix (NGTDM) Features, and Gray Level Features (GLDM) Features. The detailed definition methods of all features can be found at https://pyradiomics.readthedocs.io/en/latest/features.html, and all defined features comply with the Imaging Biomarker Standardization Initiative (IBSI) (25).

### Feature preprocessing and screening

The radiomic features of VOI delineated by different physicians were assessed for consistency using the Interclass correlation coefficients method (ICC) (26). Variables with an agreement greater than 0.75 between groups were considered reliable imaging features. Subsequently, the features were normalized using the z-score method. To identify radiomic features associated with lymph node metastasis, we performed a t-test between the negative and positive lymph node metastasis groups. Variables with a p-value< 0.05 were retained for further screening (27). Furthermore, to address collinearity among variables, Pearson correlation analysis was conducted to examine variable correlations. In cases where correlations exceeded 0.9, one of the paired variables was randomly chosen for model development. Additionally, the LASSO classifier was employed to mitigate collinearity and screen variables relevant to lymph node metastasis. Variables with non-zero coefficients in the LASSO model were ultimately used in subsequent machine learning model construction (28).

### Model construction

We utilized the presence or absence of lymph node metastasis as the target for prediction, with the characteristics obtained through the aforementioned screening strategy serving as covariates. The model was developed using the Scikit-learn (29) framework. For the intratumoral radiomics feature model, we benchmarked several algorithms, including Support Vector Machines (SVM), K-Nearest Neighbor (KNN), Random Forests, Extremely Randomized Trees (ExtraTrees), XGBoost, Light Gradient Boosting Machine (LightGBM), Multi-Layer and Perceptron (MLP), to select the best algorithm. Performance indicators such as Accuracy, area under the curve (AUC), Sensitivity, Specificity, Positive Predictive Value (PPV) and Negative Predictive Value (NPV) were calculated based on the prediction results of each model. After evaluating the combined performance of the models on the training and test sets, the MLP model was determined to be the most suitable. To ensure model comparability, MLP models were also constructed for subsequent intratumoral-peritumoral combined models and clinical feature models.

### Model performance comparison

The DeLong test was employed to compare the AUC values between different models. Additionally, the model accuracy, sensitivity, specificity, precision, recall, NPV (Negative Predictive Value), PPV (positive predictive value) and F1 score were also used for evaluating. The Decision Curve Analysis was conducted to assess the potential clinical benefit performance of the model.

### Nomogram construction

The nomogram was constructed using the R-based rms package. We integrated the radiomics model scores and clinical risk factors for lymph node metastasis and used logistic regression formulas to construct the final nomogram. The DeLong test was used to evaluate the performance of the AUC of the nomogram compared to other models. Calibration curve and DCA curve are used to evaluate the clinical benefit performance of the nomogram.

## Results

### Clinical characteristics of patients

This study enrolled 247 patients with surgically resectable NSCLC, and their chest CT images, and clinical information were collected. All patients underwent lobectomy or segmentectomy with systematic lymph node dissection. Among them, 87 patients had lymph node metastasis confirmed by postoperative pathology, while 160 patients had no lymph node metastasis. **Table 1** presents the clinical information of all included patients. Significant differences were observed between the lymph node metastasis group and the group without lymph node metastasis in terms of maximum tumor diameter (*p*<0.001) and smoking history (p<0.001). The total of 247 patients were randomly divided into training and validation cohorts, consisting of 172 and 75 patients respectively, following a 7:3 ratio. There was no statistical difference in various clinical information between the training group and the validation group (**Table 2**).

**Table 1:** Clinical characteristics of patients.

Covariates		All (n=247)	Without lymph node metastasis (n=160)	Lymph node metastasis (n=87)	*p*-value
**Age**		59.99 ± 9.06	59.9 4± 9.31	60.08 ± 8.64	0.91
**Gender (%)**	Female	130 (52.6)	89 (55.6)	41 (47.1)	0.252
	Male	117 (47.4)	71 (44.4)	46 (52.9)	
**Location (%)**	LLL*	43 (17.4)	27 (16.9)	16 (18.4)	0.525
	LUL*	65 (26.3)	41 (25.6)	24 (27.6)	
	RLL*	55 (22.3)	33 (20.6)	22 (25.3)	
	RML*	13 (5.3)	11 (6.9)	2 (2.3)	
	RUL*	71 (28.7)	48 (30.0)	23 (26.4)	
**Histology (%)**	LUAD†	203 (82.2)	134 (83.8)	69 (79.3)	0.486
	LUSC†	44 (17.8)	26 (16.2)	18 (20.7)	
**Maximum Tumor Diameter (mean ± SD)**		25.11 ± 15.43	20.16 ± 14.30	34.20 ± 13.18	<0.001
**Smoking History (%)**	No	130 (52.6)	103 (64.4)	27 (31.0)	<0.001
	Yes	117 (47.4)	57 (35.6)	60 (69.0)	

^*^LLL, Left Lower Lobe; LUL, Left Upper Lobe; RLL, Right Lower Lobe; RML, Right Middle Lobe; RUL, Right Upper Lobe. ^†^LUAD, Lung Adenocarcinoma; LUSC, Lung Squamous Cell Carcinoma.

**Table 2:** Clinical characteristics of training and testing sets.

Covariates		All (n=247)	Testing set (n=75)	Training set (n=172)	*p*-value
**Age**		59.99 ± 9.06	60.93 ± 8.83	59.58 ± 9.16	0.282
**Gender (%)**	Female	130 (52.6)	39 (52.0)	91 (52.9)	1
	Male	117 (47.4)	36 (48.0)	81 (47.1)	
**Location (%)**	LLL*	43 (17.4)	10 (13.3)	33 (19.2)	0.752
	LUL*	65 (26.3)	20 (26.7)	45 (26.2)	
	RLL*	55 (22.3)	19 (25.3)	36 (20.9)	
	RML*	13 (5.3)	5 (6.7)	8 (4.7)	
	RUL*	71 (28.7)	21 (28.0)	50 (29.1)	
**Histology (%)**	LUAD†	203 (82.2)	62 (82.7)	141 (82.0)	1
	LUSC†	44 (17.8)	13 (17.3)	31 (18.0)	
**Maximum Tumor Diameter (mean ± SD)**		25.11 ± 15.43	24.80 ± 15.38	25.24 ± 15.50	0.838
**Smoking History (%)**	No	130 (52.6)	37 (49.3)	93 (54.1)	0.584
	Yes	117 (47.4)	38 (50.7)	79 (45.9)	
**Lymph node metastasis (%)**	No	160 (64.8)	44 (58.7)	116 (67.4)	0.237
	Yes	87 (35.2)	31 (41.3)	56 (32.6)	

^*^LLL, Left Lower Lobe; LUL, Left Upper Lobe; RLL, Right Lower Lobe; RML, Right Middle Lobe; RUL, Right Upper Lobe. ^†^LUAD, Lung Adenocarcinoma; LUSC, Lung Squamous Cell Carcinoma.

### Feature extraction of intratumoral radiomics and feature selection

**Figure 1:**
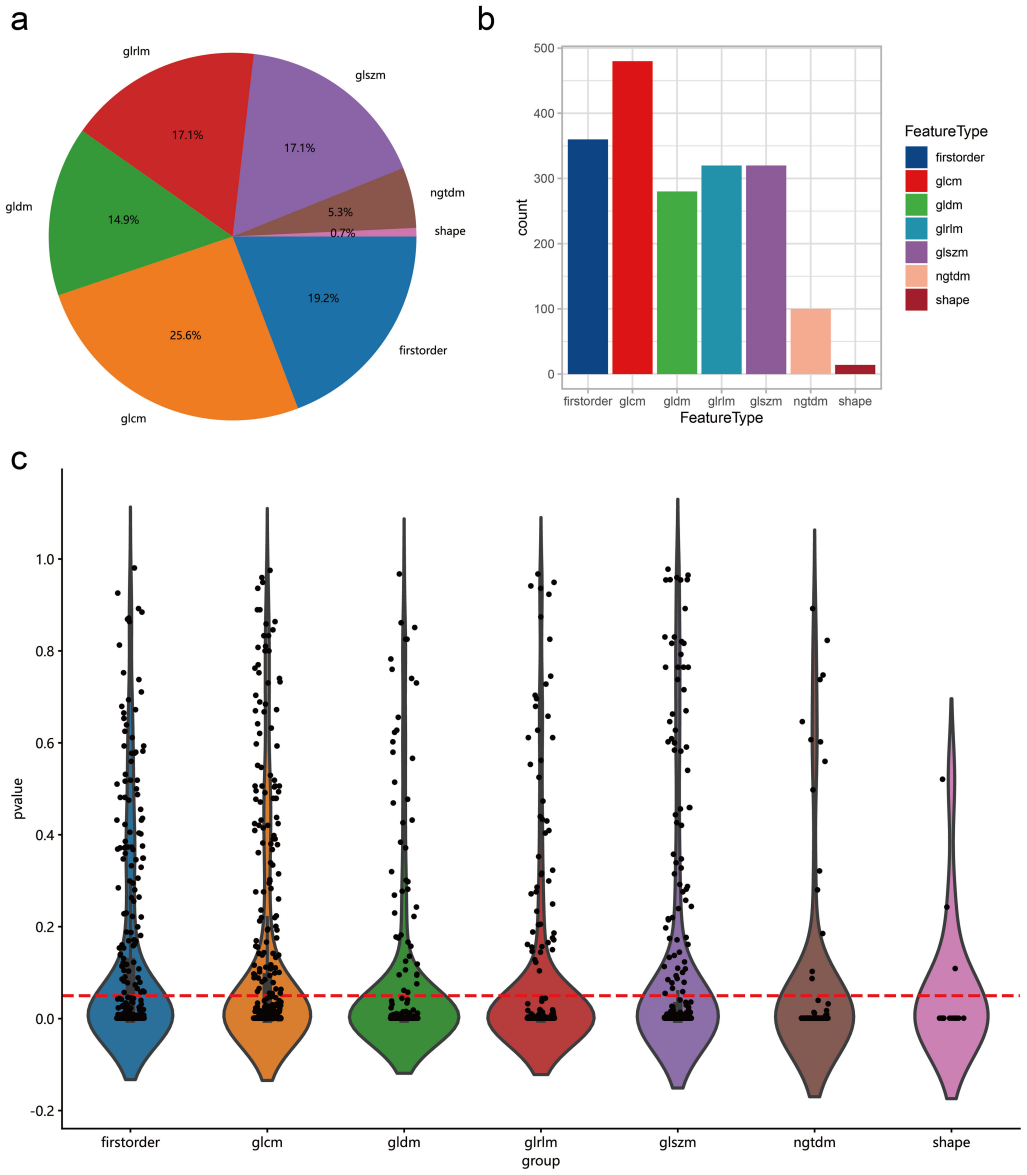
Distribution of intratumoral radiomics features data. **(A)** Proportions of different types of radiomic features. **(B)** Number of different types of radiomics features. **(C)** T-test results comparing different types of radiomic features between lymph node metastasis and non-metastasis groups. The horizontal axis represents different feature types, and the vertical axis represents p-values. Features below the red dashed line indicate a p-value <0.05.

After performing tumor region segmentation on the collected chest CT images of all patients (details provided in the Methods section), a total of 1874 intratumoral radiomic features were extracted using different filters. These features included 360 First Order Features, 14 Shape Features, 480 GLCM Features, 320 GLSZM Features, 320 GLRLM Features, 100 NGTDM Features, and 280 GLDM Features (**Figures 1A, B**). Subsequently, we performed statistical analysis between the groups with and without lymph node metastasis to screen for relevant radiomics features. We retained 1383 features with a *p*-value < 0.05 for further analysis (**Figure 1C**). To address collinearity among variables, Pearson correlation analysis was conducted on these 1383 variables. Based on the experience of previously published articles (30), for variables with correlations greater than 0.9, one variable was randomly retained, resulting in 271 variables being finally retained.

### Model construction of intratumoral radiomic features

**Figure 2:**
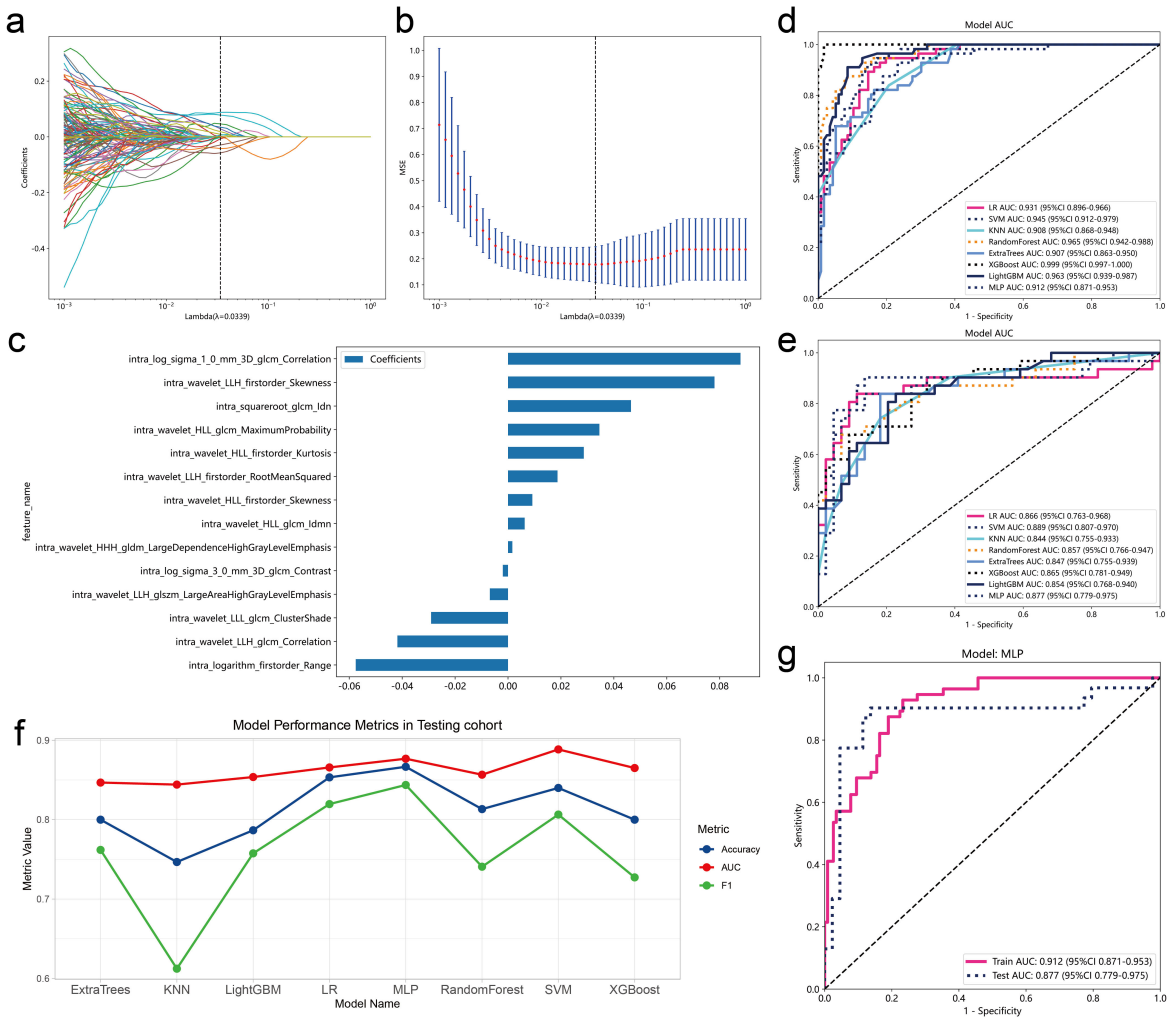
Screening and model construction of intratumoral radiomics features. The LASSO classifier is used for feature selection. **(A)** Coefficient trajectory plot for various Lambda values. **(B)** Mean squared error (MSE) of the model at different Lambda values. The black dashed line indicates the optimal Lambda value determined by minimizing the MSE. **(C)** Coefficient plot for variables in the model at the optimal Lambda value. The X-axis represents model coefficients. Model metrics **(D)** and ROC curves **(E, F)** of the model on the training and testing sets of different machine learning models. **(G)** ROC curve of the MLP model, determined as the optimal model, on the training and testing sets.

In the training group, we employed the LASSO classifier to further identify variables that significantly influenced lymph node metastasis for constructing subsequent models. When λ reaches the optimal value of 0.0339, fourteen variables with non-zero coefficients were retained for subsequent modeling (**Figures 2A–C**). Various machine learning algorithms including SVM, KNN, Random Forests, ExtraTrees, LightGBM, and MLP were benchmarked to select the best algorithm. All models underwent 5-fold cross-validation in entire dataset. In 5-fold cross-validation, the MLP model showed the highest median AUC (**Supplementary Figure 1A**). Therefore, the MLP model may be a potentially optimal model. In subsequent modeling, we use the training set for training and the testing set to evaluate the final metrics of the model. (**Table 3**). The accuracy, AUC values and F1 scores of the models were visualized for testing set (**Figures 2D–F**). It is worth noting that among all models, although SVM has the highest AUC, the MLP model has higher accuracy and F1 Score. Since there is a certain imbalance between the two categories in our data, F1 scores is more robust to evaluating the performance of the model than AUC. Therefore, we chose the MLP model as the final model (**Figure 2G**). The MLP model constructed using intratumoral radiomic features achieved AUC values of 0.912 (95% CI: 0.871-0.953) and 0.877 (95% CI: 0.779-0.975) in the training and testing sets, respectively, for predicting lymph node metastasis.

**Table 3:** Model performance of intratumoral model.

Model_name	Accuracy	AUC	95% CI	Sensitivity	Specificity	PPV^*^	NPV^*^	Precision	Recall	F1	Cohort
LR	0.843	0.931	0.8956 - 0.9661	0.929	0.802	0.693	0.959	0.693	0.929	0.794	Train
	0.853	0.866	0.7633 - 0.9684	0.806	0.886	0.833	0.867	0.833	0.806	0.820	Test
SVM	0.860	0.945	0.9118 - 0.9789	0.929	0.828	0.722	0.960	0.722	0.929	0.812	Train
	0.840	0.889	0.8071 - 0.9700	0.806	0.864	0.806	0.864	0.806	0.806	0.806	Test
KNN	0.814	0.908	0.8684 - 0.9482	0.571	0.931	0.800	0.818	0.800	0.571	0.667	Train
	0.747	0.844	0.7551 - 0.9333	0.484	0.932	0.833	0.719	0.833	0.484	0.612	Test
RandomForest	0.872	0.965	0.9418 - 0.9880	0.929	0.845	0.743	0.961	0.743	0.929	0.825	Train
	0.813	0.857	0.7662 - 0.9471	0.645	0.932	0.870	0.788	0.870	0.645	0.741	Test
ExtraTrees	0.826	0.907	0.8635 - 0.9498	0.804	0.836	0.703	0.898	0.703	0.804	0.750	Train
	0.800	0.847	0.7547 - 0.9388	0.774	0.818	0.750	0.837	0.750	0.774	0.762	Test
XGBoost	0.983	0.999	0.9965 - 1.0000	0.982	0.983	0.965	0.991	0.965	0.982	0.973	Train
	0.800	0.865	0.7810 - 0.9492	0.645	0.909	0.833	0.784	0.833	0.645	0.727	Test
LightGBM	0.907	0.963	0.9394 - 0.9870	0.893	0.914	0.833	0.946	0.833	0.893	0.862	Train
	0.787	0.854	0.7675 - 0.9399	0.806	0.773	0.714	0.850	0.714	0.806	0.758	Test
MLP	0.814	0.912	0.8710 - 0.9532	0.911	0.767	0.654	0.947	0.654	0.911	0.761	Train
	0.867	0.877	0.7790 - 0.9746	0.871	0.864	0.818	0.905	0.818	0.871	0.844	Test

^*^NPV, Negative Predictive Value; PPV, positive predictive value.

### Radiomics feature extraction and feature selection for intratumoral and peritumoral combination

**Figure 3:**
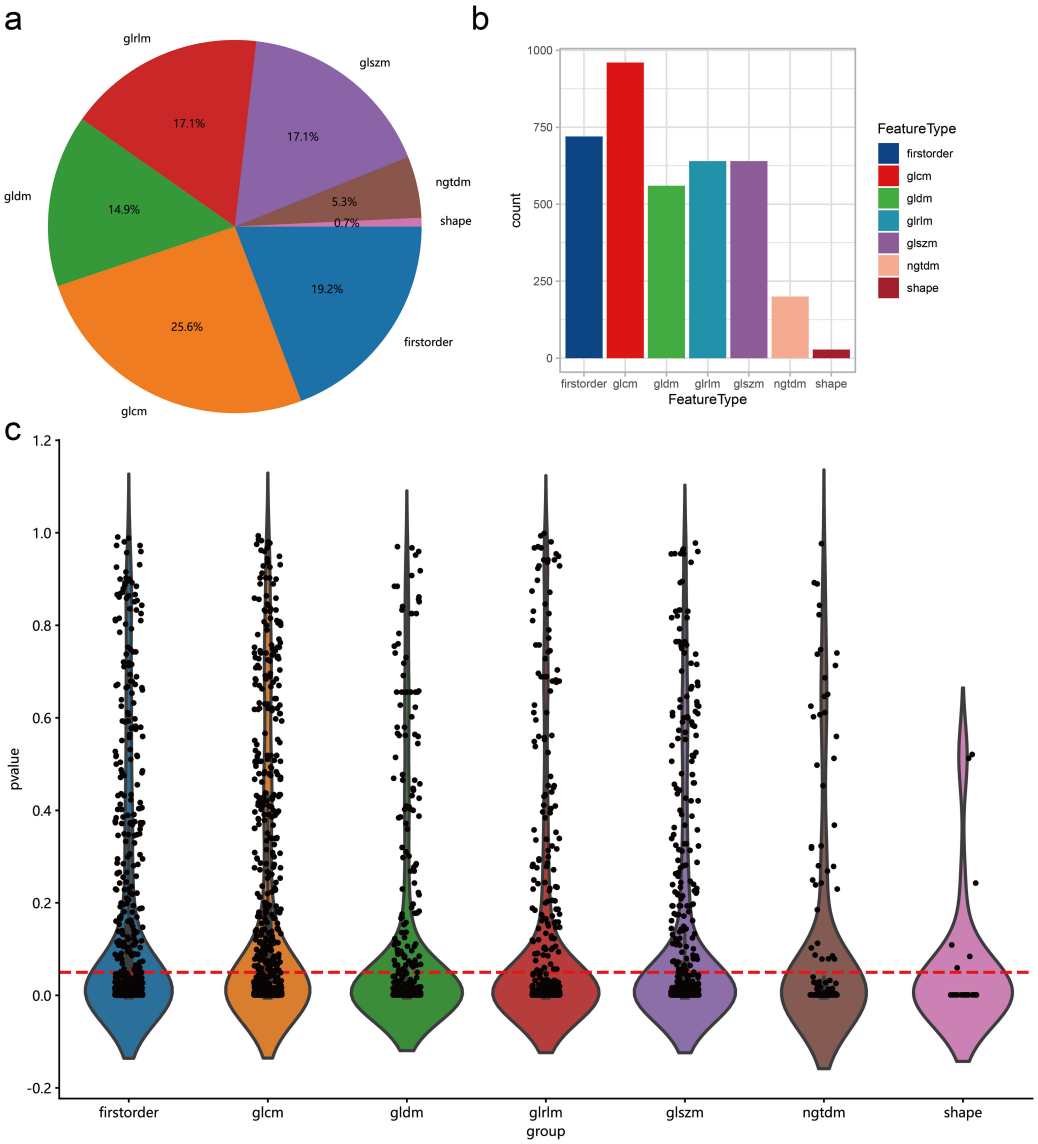
Distribution of intratumoral and peritumoral radiomics features data. **(A)** Proportions of different types of radiomic features. **(B)** Number of different types of radiomics features. **(C)** T-test results comparing different types of radiomic features between lymph node metastasis and non-metastasis groups. The horizontal axis represents different feature types, and the vertical axis represents p-values. Features below the red dashed line indicate a p-value <0.05.

To investigate the impact of peritumoral radiomics features on the predictive ability of lymph node metastasis, we extended manually delineated tumor ROI regions outwards by 3 individual voxels to obtain peritumoral regions by referring to methods in previously published articles (8, 31). Peritumoral features were extracted using the same method as intratumoral feature extraction and merged with intratumoral features for subsequent feature selection. After combination, we obtained a total of 3748 intratumoral and peritumoral radiomics features. Similarly, we screened and obtained 2572 features with *p*-value < 0.05 in lymph node metastasis and non-lymph node metastasis samples (**Figures 3A–C**). As in the modeling of intratumoral features only, we performed Pearson correlation analysis on these combined features and retained one variable from paired variables whose correlation between variables was greater than 0.9. Eventually, we selected 539 variables for subsequent construction of intratumoral and peritumoral prediction models.

### Model construction of intratumoral and peritumoral combination radiomic features

**Figure 4:**
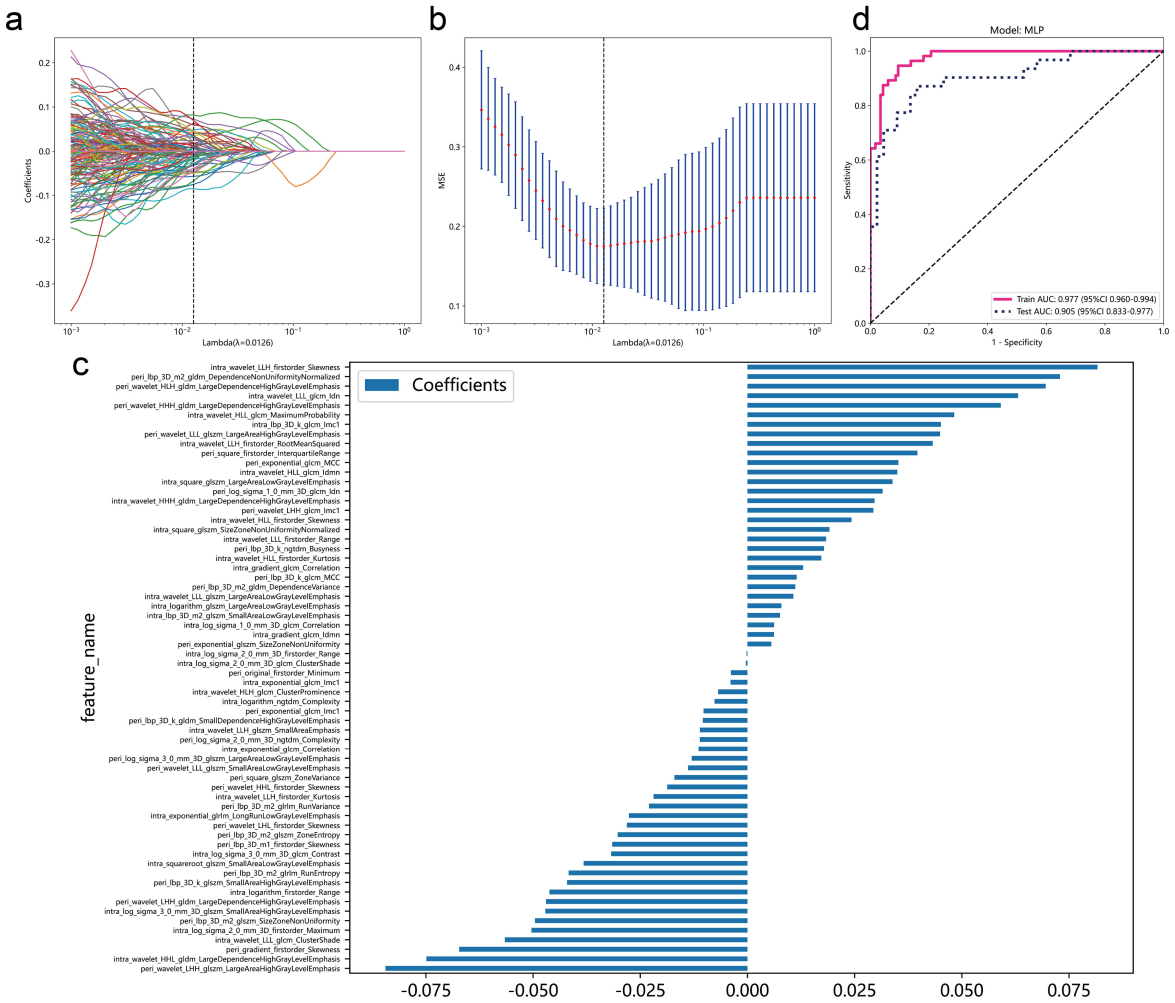
Screening and model construction of intratumoral and peritumoral radiomics features. The LASSO classifier is used for feature selection. **(A)** Coefficient trajectory plot for various Lambda values. **(B)** Mean squared error (MSE) of the model at different Lambda values. The black dashed line indicates the optimal Lambda value determined by minimizing the MSE. **(C)** Coefficient plot for variables in the model at the optimal Lambda value. The X-axis represents model coefficients. **(D)** ROC curve of the MLP model on the training and testing sets.

Consistent with the modeling strategy using only intratumoral features, we employed the LASSO classifier to screen combined intratumoral and peritumoral features and used the MLP algorithm to test the impact of peritumoral features on the model for variables with absolute values of coefficients greater than zero. After LASSO classifier screening, we finally obtained 64 variables with non-zero coefficients (**Figures 4A–C**). Using the MLP algorithm to predict lymph node metastasis status, AUC values of the model reached 0.977 (95%CI: 0.960-0.994) in the training set and 0.905 (95%CI: 0.833-0.977) in the testing set (**Figure 4D**). In addition, since the SVM model of intratumoral radiomics features showed the highest accuracy (**Figure 2F**), we also tried the SVM modeling after combining intratumoral and peritumoral features. In the test group, the SVM model of intratumoral and peritumoral features had an AUC of 0.876 (95% CI 0.792 – 0.960) (**Supplementary Figure 2A**), which was lower than the MLP model. In addition, the F1 score of the SVM model in testing set is also lower than that of the MLP model (**Table 4**). Finally, the MLP model was identified as the suitable model of intratumoral and peritumoral features. Overall, compared to the AUC in the MLP model when using only intratumoral features (**Figure 2G**), the predictive performance of the MLP model improved to a certain extent upon inclusion of peritumoral features. In addition, we validated the intratumor-peritumoral feature MLP model in an external dataset. The AUC of the external dataset was 0.812 (95% CI, 0.741-0.884), which showed that the model had good robustness (**Supplementary Figure 3A**).

**Table 4:** Model performance of intratumoral-peritumoral model.

Model_name	Accuracy	AUC	95% CI	Sensitivity	Specificity	PPV^*^	NPV^*^	Precision	Recall	F1	Cohort
SVM	0.948	0.993	0.9851 - 1.0000	0.946	0.948	0.898	0.973	0.898	0.946	0.922	Train
	0.827	0.876	0.7921 - 0.9601	0.774	0.864	0.800	0.844	0.800	0.774	0.787	Test
MLP	0.913	0.977	0.9595 - 0.9937	0.929	0.905	0.825	0.963	0.825	0.929	0.874	Train
	0.840	0.905	0.8327 - 0.9766	0.839	0.841	0.788	0.881	0.788	0.839	0.812	Test

^*^NPV, Negative Predictive Value; PPV, positive predictive value.

### Clinical model construction

**Figure 5:**
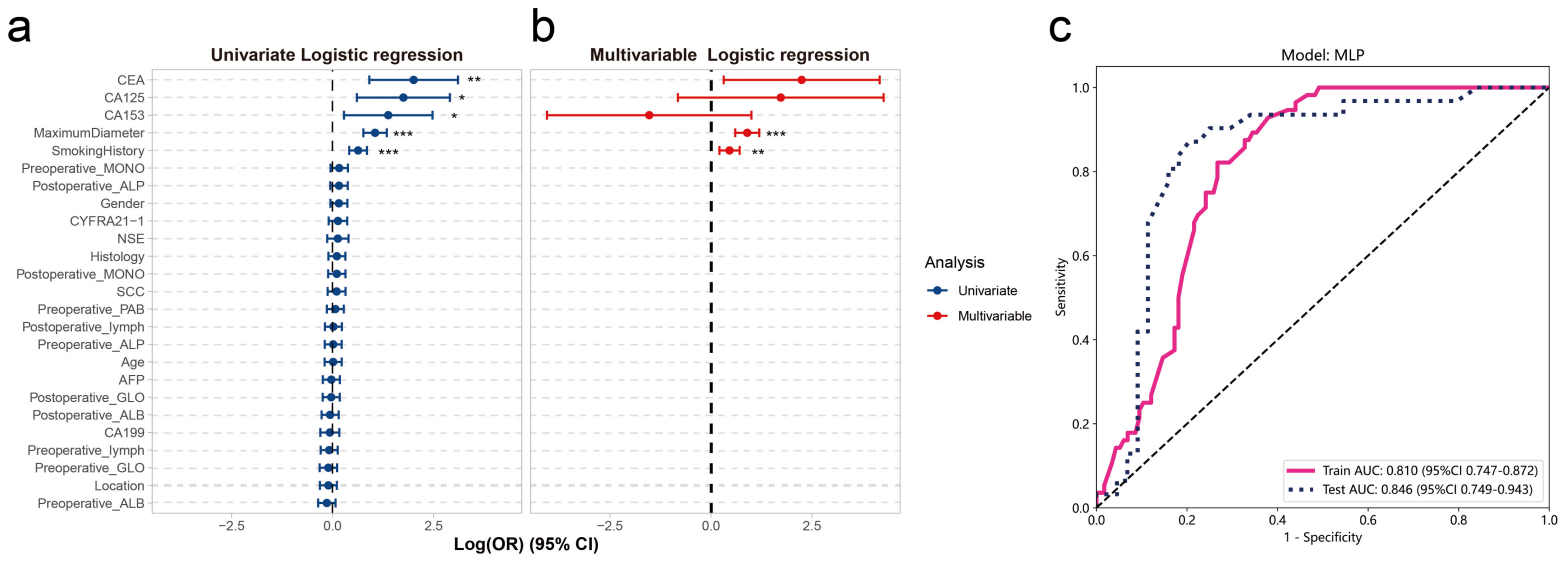
Clinical feature selection and model construction. Univariate **(A)** and multivariate **(B)** logistic regression of clinical features, with the x-axis representing log2-transformed odds ratios. **(C)** ROC curve of the MLP model for clinical features. In **(A, B)** “*” indicates *P*-value < 0.05, “**” indicates *P*-value < 0.01, and “***” indicates *P*-value < 0.001.

Clinical information as an important indicator of current clinical assessment, we collected patients’ clinical information, including age, gender, preoperative CT diagnosis of tumor location, smoking history, maximum tumor diameter, pathological types of tumors diagnosed by intraoperative frozen sections and a series of laboratory indicators. Univariate logistic regression was applied to these clinical details to assess their risks for lymph node metastasis. Results indicated that maximum tumor diameter, smoking history, CA153, CA125 and CEA were risk factors for lymph node metastasis (**Figure 5A**). Additionally, we conducted multivariate logistic regression on these risk factors. In the multivariate analysis, smoking history and maximum tumor diameter were considered independent risk factors for lymph node metastasis (**Figure 5B**, **Supplementary Table 1**). To ensure the comparability of the model, we also used the MLP method to construct a lymph node metastasis prediction model using these independent risk factors, and the results showed that the clinical information MLP model had an AUC of 0.810 (95%CI: 0.747-0.872) and 0.846 (95%CI: 0.749-0.943) in the training and validation groups, respectively (**Figure 5C**).

### Model performance evaluation

**Figure 6:**
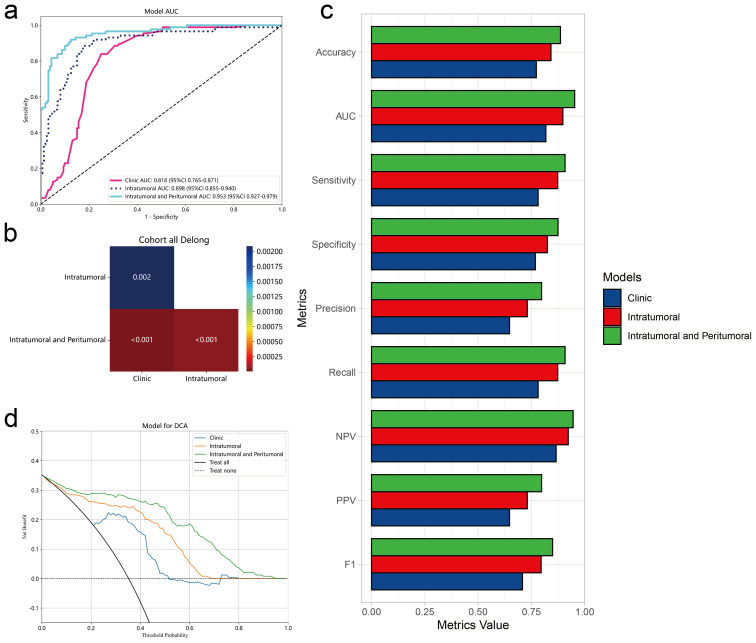
Comparison of multiple model performance. **(A)** ROC curves of the clinical feature model, intratumoral radiomic feature model, and combined intratumoral and peritumoral radiomic feature model in the entire dataset. **(B)** DeLong test for AUC values of the ROC curves of the three models. **(C)** The comparison of all performance metrics of the three models. **(D)** Decision Curve Analysis (DCA) curves of the three models in the entire dataset.

To evaluate the performance of the three models we constructed above (intratumoral peritumoral radiomics model, intratumoral radiomics model and clinical information model), we compared various model indicators in the entire data set. Radiomic signatures of intratumoral and peritumoral regional features still exhibit higher area under the ROC curve than intratumoral features or clinical feature models alone and DeLong test shows that p values are all <0.05 (**Figures 6A, B**). Furthermore, when we summarized other evaluation metrics for the three models, we found that the intratumoral and peritumoral feature models were overall higher than other models in terms of accuracy, sensitivity, specificity, precision, recall, NPV (Negative Predictive Value), PPV (positive predictive value) and F1 score (**Figure 6C**, **Table 5**). Clinical decision curves further demonstrated that the model combining intratumoral and peritumoral radiomics features potentially offers greater clinical benefits (**Figure 6D**). In order to further evaluate the stability of the predictive performance of the three models across the entire dataset, we adopted 5-fold cross-validation. The results demonstrate that the model incorporating both intratumoral and peritumoral features still exhibits the highest average AUC value. (**Supplementary Figures 4A–C**) In summary, incorporating peritumoral radiomic features into the prediction model improves the performance of the model in predicting lymph node metastasis and may provide potential clinical benefits. Therefore, in this study the results show that including peritumoral features in radiological models is necessary for predicting lymph node metastasis.

**Table 5:** Model metrics comparison.

Model name	Accuracy	AUC	95% CI	Sensitivity	Specificity	PPV*	NPV*	Precision	Recall	F1	Cohort
Clinic	0.773	0.818	0.7653 - 0.8709	0.782	0.769	0.648	0.866	0.648	0.782	0.708	Entire
Intratumoral	0.842	0.898	0.8553 - 0.9402	0.874	0.825	0.731	0.923	0.731	0.874	0.796	Entire
Intratumoral and Peritumoral	0.887	0.953	0.9272 - 0.9792	0.908	0.875	0.798	0.946	0.798	0.908	0.849	Entire

^*^NPV, Negative Predictive Value; PPV, positive predictive value.

### Construction of lymph node metastasis nomogram of lung adenocarcinoma

**Figure 7:**
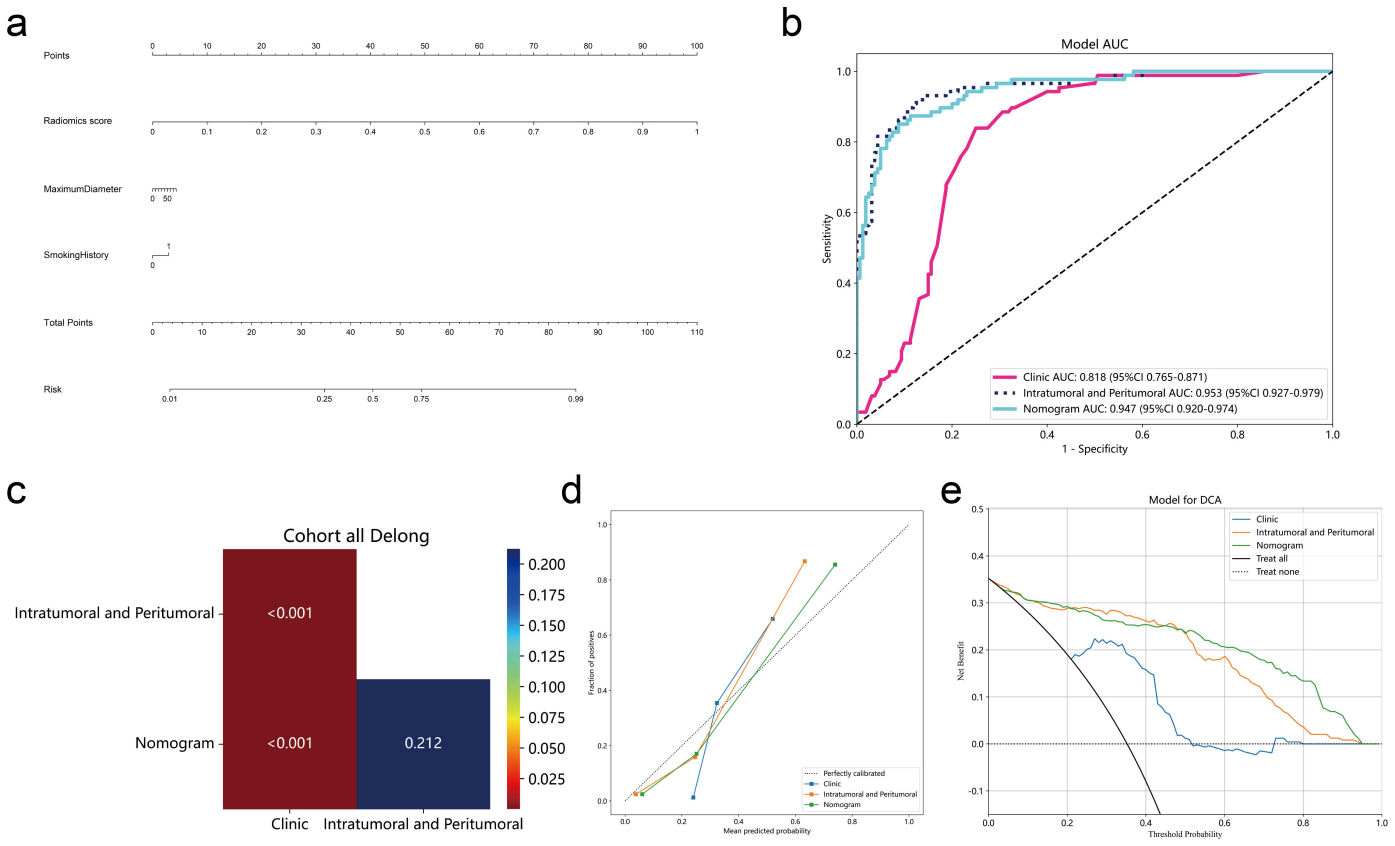
Nomogram integrating intratumoral-peritumoral radiomics model and clinical indicators. **(A)** Nomogram for predicting lymph node metastasis in non-small cell lung cancer. **(B)** The ROC curve of nomogram, intratumoral-peritumoral radiomics model and clinical model. **(C)** DeLong test of AUC values between different models. **(D, E)** Calibration curves and Decision Curve Analysis of different models.

Because of the importance and usefulness of clinical information in clinical assessment, we should consider it. Therefore, we further constructed a nomogram by integrating intratumoral peritumoral radiomics models and clinical risk indicators of lymph node metastasis to increase clinical applicability (**Figure 7A**). The AUC of the nomogram in the entire cohort was 0.947 (95% CI 0.920-0.974) (**Figure 7B**). DeLong test showed that the AUC of the nomogram was higher than the clinical features model (p<0.001) and comparable to the intratumoral peritumoral radiomics model (p=0.212) (**Figure 7C**). The calibration curves and Decision Curve Analysis still show that the nomogram has comparable performance to the intratumoral peritumoral radiomics model and may bring potential clinical benefit (**Figures 7D, E**).

## Discussion

Accurate clinical staging is crucial for determining treatment strategies in newly diagnosed NSCLC patients, particularly for surgically resectable patients. Accurate N staging can guide decisions regarding preoperative neoadjuvant therapy (32) or intraoperative lymph node dissection strategies (33). Radiomics is a promising non-invasive diagnostic approach for lymph node N staging compared to mediastinoscopic lymph node biopsy or endobronchial ultrasound-guided fine needle aspiration. At present, lymph node staging often relies on intraoperative sampling. However, the diagnosis based on preoperative radiomics is promising. In this study, we established a MLP model combining the intratumoral and peritumoral radiomics characteristics of NSCLC on CT images, which was superior to traditional intratumoral feature models and clinical information models in predicting lymph node metastasis status preoperatively. In addition, the Nomogram integrating clinical information and intratumoral-peritumoral radiomics still have a good predictive ability.

Although machine learning models based on intratumoral radiomics profiles have proved effective in predicting lymph node metastasis status (16, 34, 35), peritumoral profiles have received limited attention in research. The peritumoral area provides insights into tumor infiltration and the invasion of microvessels and lymphatic vessels, making the radiomic characteristics of this region crucial (36, 37). Additionally, manual segmentation and measurement of the tumor area often result in unstable features at the tumor edge. Due to the irregular shape of the tumor edge area, parts of the tumor margin area will inevitably be missing due to manual division. Therefore, expanding the artificially segmented tumor area to include the peritumoral region partially addresses this issue to enhance model generalization ability. In addition, the texture changes, abnormal local blood vessel density, and information on the degree of tumor infiltration provided by the surrounding tissue characteristics also enable the model to evaluate the invasiveness and metastasis potential of the tumor from a more comprehensive perspective, thus enhancing the performance of the model. We found an article on intratumoral and peritumoral radiomics profiles for predicting lymph node metastasis status, it focused exclusively on patients with stage IA NSCLC (38). However, it is necessary to include surgically resectable cases ranging from stage IA to stage IIIA, encompassing only localized lymph node metastases. Compared with previously published models for predicting lymph nodes based on PET/CT (39, 40), CT is the first choice examination for patients with initial treatment of pulmonary nodules, may make our model more universal. In addition, PET/CT relies on FDG (fluorodeoxyglucose) uptake imaging, which may be affected by infectious or non-infectious diseases (such as tuberculosis, pneumoconiosis, or chronic obstructive pulmonary disease. In our study, we developed three prediction models for lymph node metastasis status in all included patients with surgically resectable NSCLC: an intratumoral radiomics profile model, a combined peritumoral and intratumoral radiomics profile model, and a clinical information prediction model. Upon comparing their performance, we observed that models incorporating peritumoral radiomics profiles was better in each metric (accuracy:0.887, sensitivity:0.908, specificity:0.875, precision:0.798, recall:0.908, negative predictive value:0.946, positive predictive value:0.798 and F1 score:0.849). Fusion radiomics features and clinical information making our model more applicable. Our constructed nomogram can predict lymph node metastasis of lung cancer in a non-invasive manner, which is of great significance for clinical treatment planning of lung cancer patients. Our results found that incorporating radiomic features of the peritumoral region is necessary in the prediction of lymph node metastasis in non-small cell lung cancer. The information it provides on texture changes, abnormal local blood vessel density, and the extent of tumor infiltration is indispensable for clinical assessment of the invasiveness and metastatic potential of primary tumors. Comprehensive consideration of the characteristics of the tumor itself and the characteristics of these surrounding areas can enhance the accuracy of lymph node metastasis prediction, bringing potential clinical benefits to patients.

This study also has some limitations. Even though we used a publicly available dataset for external validation, there is still a lack of multicenter data to further verify the generalization ability of the model. In addition, the dataset in this study also has the problem of imbalance in the number of outcome categories. To eliminate the impact of class imbalance on model evaluation as much as possible, we not only focus on the accuracy of the model, but also compare the AUC value under binary classification between models and the F1 score calculated by precision and recall. In addition, the error caused by manual segmentation of ROI areas should also be considered. Although we performed Intraclass Correlation Coefficient analysis after segmentation by multiple doctors to screen for robust radiomics features, this issue has not been completely resolved. In future studies, we would collect multicenter data for validation and establish better deep learning algorithm models to solve feature biases caused by manual segmentation of tumor regions. Furthermore, in the practical application of radiomics models, how to ensure the standardization and automation of the preprocessing process of images from different hospitals or different CT scanners is also a problem that needs to be solved urgently.

## Conclusion

Our study proposed a predictive model for lymph node metastasis status based on preoperative CT radiomics profiles in patients with operable resected NSCLC. This model included the characteristics of peritumoral region and improved the performance of traditional intratumoral feature models and clinical feature models. Additionally, our constructed nomogram may provide new insights into the development of clinical treatment strategies for patients with surgically resectable NSCLC. Our results show that radiomics models considering the characteristics of the tumor region alone are insufficient and future studies should pay more attention to the region surrounding the tumor.

## Data Availability

The original contributions presented in the study are included in the article/**Supplementary Material**. Further inquiries can be directed to the corresponding author.
