# Supplementary material for: Evaluating peritumoral and intratumoral radiomics signatures for predicting lymph node metastasis in surgically resectable non-small cell lung cancer
Source: Front Oncol. 2024 Oct 11;14:1427743. doi: 10.3389/fonc.2024.1427743 (PMC11502299; doi:10.3389/fonc.2024.1427743)
Supplement: Supplementary file 1 [file DataSheet1.docx]

Supplementary Material

# Supplementary Figures

**
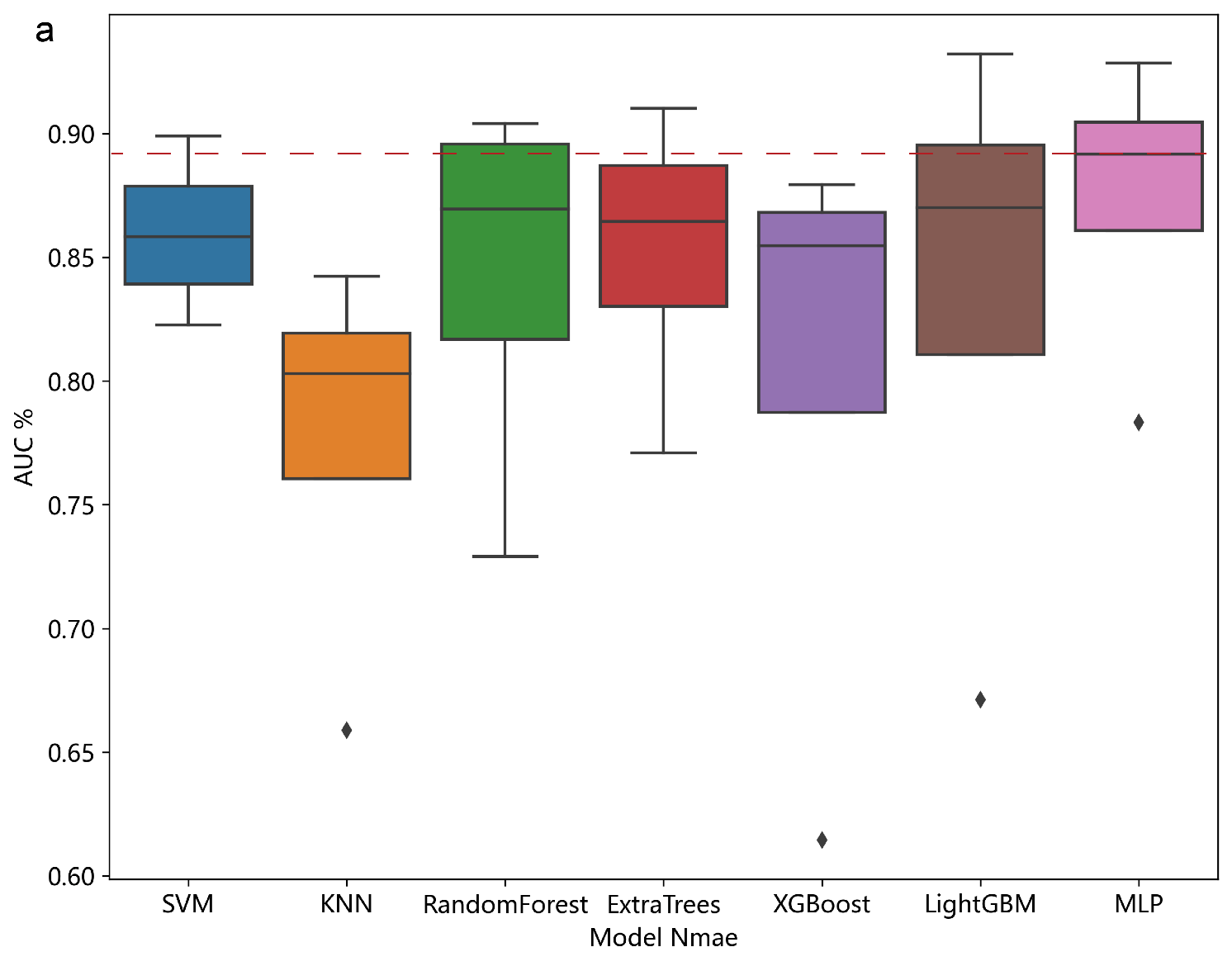
**

**Supplementary Figure 1.** (a) 5-fold cross-validation of the models.


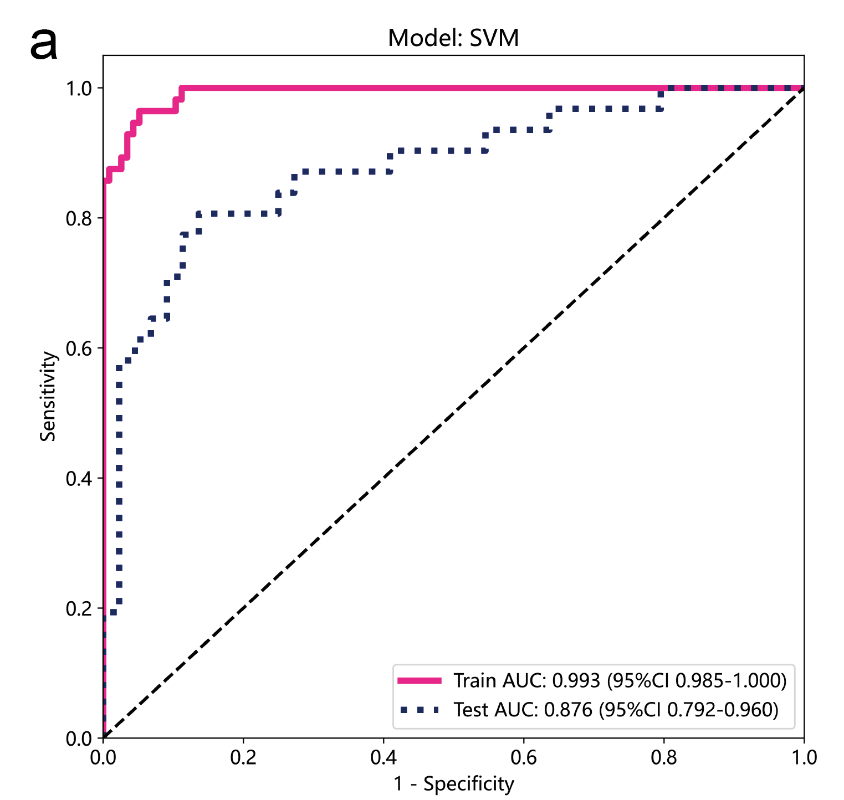


**Supplementary Figure 2.** (a) The ROC curve of SVM model constructed using intratumoral and peritumoral radiomic features.


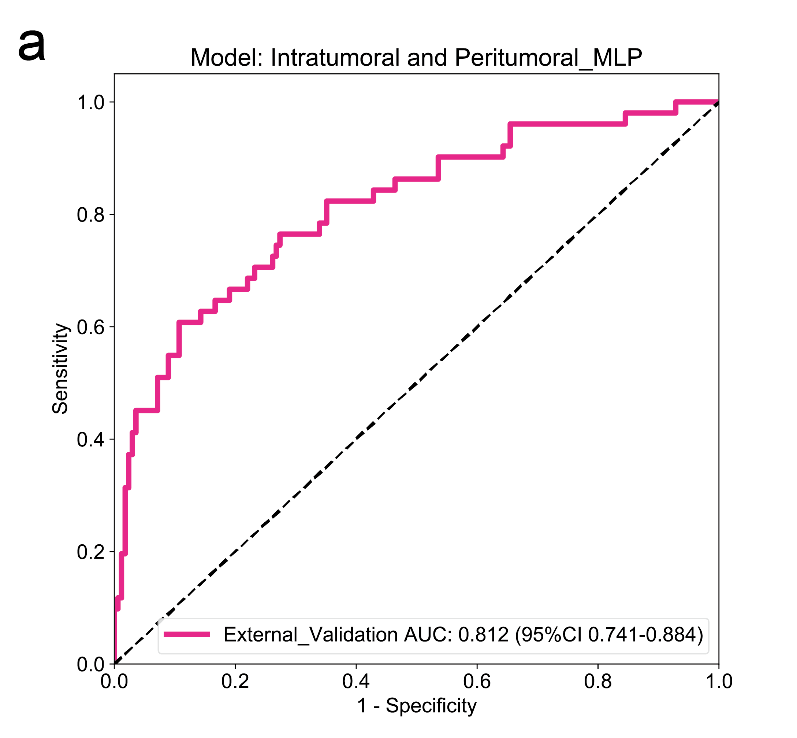


**Supplementary Figure 3.** (a) The external dataset validation ROC curve for the intratumoral and peritumoral radiomics features model.


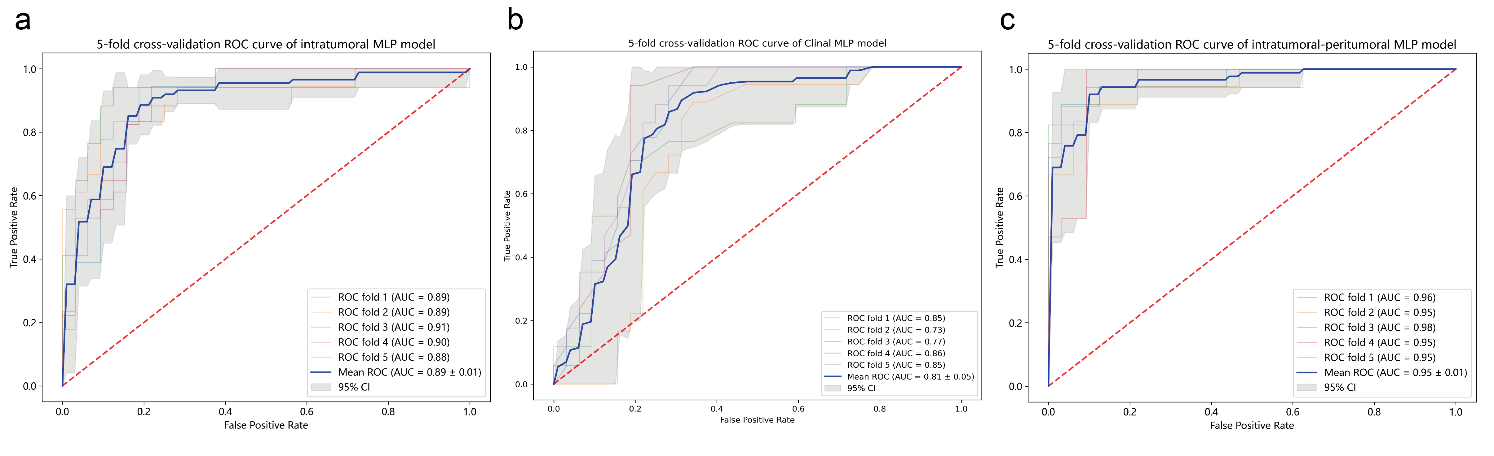


**Supplementary Figure 4.** 5-fold cross-validation of the three MLP models in entire dataset (a) Intratumoral radiomics feature model (b) Clinical feature model (c) Intratumoral-peritumoral radiomics feature model.

Supplementary Table 1. Univariate and multivariate logistic regression analysis for screening risk factors for lymph node metastasis in lung adenocarcinoma

| **Features_Name** | **Univariate Logistic Regression** | | **Multivariate Logistic Regression** | |
| --- | --- | --- | --- | --- |
|  | OR(95%CI) | *P*-value | OR(95%CI) | *P*-value |
| Preoperative_ALB*** | 0.867(0.7-1.075) | 0.274 | - | - |
| Location | 0.901(0.731-1.112) | 0.416 | - | - |
| Preoperative_GLO*** | 0.901(0.726-1.117) | 0.424 | - | - |
| Preoperative_lymph*** | 0.92(0.745-1.137) | 0.515 | - | - |
| CA199 | 0.934(0.738-1.182) | 0.632 | - | - |
| Postoperative_ALB*** | 0.94(0.761-1.162) | 0.632 | - | - |
| Postoperative_GLO*** | 0.966(0.783-1.192) | 0.787 | - | - |
| AFP | 0.969(0.785-1.196) | 0.807 | - | - |
| Age | 1.014(0.822-1.251) | 0.914 | - | - |
| Preoperative_ALP*** | 1.015(0.823-1.251) | 0.91 | - | - |
| Postoperative_lymph*** | 1.021(0.827-1.259) | 0.873 | - | - |
| Preoperative_PAB*** | 1.07(0.867-1.32) | 0.597 | - | - |
| SCC | 1.104(0.884-1.379) | 0.463 | - | - |
| Postoperative_MONO*** | 1.11(0.897-1.374) | 0.421 | - | - |
| Histology | 1.112(0.901-1.373) | 0.406 | - | - |
| NSE | 1.139(0.875-1.483) | 0.415 | - | - |
| CYFRA21-1 | 1.142(0.908-1.435) | 0.339 | - | - |
| Gender | 1.169(0.947-1.442) | 0.223 | - | - |
| Postoperative_ALP*** | 1.172(0.944-1.455) | 0.227 | - | - |
| Preoperative_MONO*** | 1.177(0.949-1.459) | 0.213 | - | - |
| SmokingHistory | 1.879(1.508-2.342) | **<0.001** | 1.584(1.234-2.034) | **0.002** |
| MaximumDiameter | 2.861(2.143-3.823) | **<0.001** | 2.454(1.822-3.307) | **<0.001** |
| CA153 | 3.963(1.326-11.846) | **0.039** | 0.217(0.017-2.721) | 0.32 |
| CA125 | 5.764(1.824-18.229) | **0.012** | 5.629(0.441-71.808) | 0.264 |
| CEA | 7.436(2.484-22.265) | **0.003** | 9.438(1.37-65.04) | 0.056 |

**ALB: Albumin, GLO: Globulin, LYMPH: Lymphocyte count, ALP: Alkaline phosphatase, PAB: Prealbumin, MONO: Monocyte count*
